# Co-occurrence and clustering characteristics of health risk behaviors among older adults in China

**DOI:** 10.3389/fpubh.2025.1653090

**Published:** 2025-12-03

**Authors:** Pei Sun, Jie Gao, Ting Xiao, Xiao Liang, Xin Zhang, Xiao Zhang, Xiaopeng Yan, Chunping Ni

**Affiliations:** Department of Basic Nursing, School of Nursing, Air Force Medical University, Xi'an, China

**Keywords:** health risk behaviors, older adults, co-occurrence, clustering, network analysis

## Abstract

**Background:**

A considerable proportion of older adults exhibit multiple modifiable health risk behaviors. Identifying not only the co-occurrence of health risk behaviors but, more importantly, their specific clustering patterns is essential for developing targeted multiple health behavior interventions.

**Methods:**

The study sample comprised 12,766 participants from the 2018 wave of the CLHLS. Data on their health risk behaviors (inadequate sleep, insufficient fruit intake, insufficient vegetable intake, salty diet, smoking, drinking, and irregular exercise), basic demographic information, and health outcomes (self-rated quality of life scores and self-rated health status) were obtained. Analytical methods included descriptive statistics, Spearman correlation, binary logistic regression, Odds/Expected (O/E) ratios, and network community detection.

**Results:**

The median age of the participants was 84 (75, 93) years, with 5,891 (46.15%) males. A high prevalence (64.36%) of co-occurring health risk behaviors (≥2) was observed. There was a negative correlation between the number of co-occurring health risk behaviors and self-assessed quality of life (*r* = −0.173, *p* < 0.01), as well as self-assessed health level (*r* = −0.141, *p* < 0.01). Gender, age, years of education, residence, and economic status significantly influenced the co-occurrence of behaviors (all *p* < 0.01). Smoking and drinking (O/E = 2.67), insufficient fruit and vegetable intake (O/E = 1.60), and a salty diet and smoking (O/E = 1.32) demonstrated the most significant clustering risks. Two distinct clustering patterns were identified, which can be termed an Addiction Behavior Pattern and an Unhealthy Activity-Eating Pattern.

**Conclusion:**

The co-occurrence of health risk behaviors is prevalent and associated with adverse health outcomes. Behaviors exhibit a clustering effect and demonstrate distinct clustering patterns. Public health interventions should move beyond single-behavior approaches to develop integrated strategies that target these specific clustering patterns for more effective management of multiple health risks.

## 1 Introduction

China is undergoing the most rapid and large-scale population aging in the world, which poses unprecedented challenges to the public health system. Health risk behaviors, including smoking, drinking, unhealthy dietary habits, and physical inactivity, are primary determinants of health outcomes and quality of life among older adults (1, 2). In the context of an increasing public health burden, intervening in these modifiable behaviors has become one of the most cost-effective prevention and control strategies. The simultaneous occurrence of multiple health risk behaviors in an individual, known as the co-occurrence of health risk behaviors, exerts a synergistic detrimental impact on health (3, 4). The reported prevalence of this co-occurrence ranges from 37.0 to 66.9% across studies in different countries (5–8). Collectively, these data demonstrate that, while influenced by demographic characteristics and survey variables, the co-occurrence of health-risk behaviors is a prevalent issue among older adults.

In this context, traditional interventions targeting a single behavior are less suited to the needs of older adults compared to multiple health behavior change (MHBC) strategies (9, 10). It is worth noting that the co-occurrence of health risk behaviors is not randomly distributed. Current research suggests that certain health risk behaviors tend to co-occur at higher rates due to shared influencing factors or direct underlying associations, thereby demonstrating a clustering effect (11). For instance, individuals who smoke are more likely to consume alcohol. Thus, mapping these specific clustering patterns—not just quantifying co-occurrence—is critical for developing efficient and integrated interventions.

Current research on the clustering characteristics of health-risk behaviors has predominantly focused on adolescent populations (12–14). Existing evidence indicates that these clustering patterns are influenced by demographic characteristics such as sex and age. For instance, a study conducted in South Korea demonstrated distinct differences in the clustering of health-risk behaviors between men and women (15), while research by Hale et al. (16) identified variations in clustering characteristics across different age groups. Consequently, although research on the clustering characteristics of health-risk behaviors in adolescents exists, these findings cannot be directly generalized to older adults. Currently, existing research on older adults is predominantly limited to reporting the prevalence of single behaviors or merely describing the rate of multiple behavioral co-occurrence, thereby leaving the clustering patterns of health-risk behaviors in this population underexplored.

This study analyzes data from the Chinese Longitudinal Healthy Longevity Survey (CLHLS) to go beyond describing the co-occurrence of health-risk behaviors among older adults in China and to uncover their specific clustering patterns. We anticipate that these findings will provide crucial evidence for developing targeted and efficient MHBC interventions tailored to older adults.

## 2 Methods

### 2.1 Data sources

The data utilized in this study was sourced from the 2018 wave of the Chinese Longitudinal Healthy Longevity Survey (CLHLS). As a nationally representative survey covering most provinces in China, the CLHLS focuses on older adults and seeks to identify key factors affecting health and longevity (17). The interviews were carried out by trained field staff at the participants’ homes. The integrity and reliability of the data have been validated and are widely recognized in prior research (18). The CLHLS study received approval from the Research Ethics Committee of Peking University (IRB00001052-13074), and informed consent was obtained in writing from all participants or their surrogate respondents.

The 2018 wave of the CLHLS encompassed survey data from 15,874 older adults. Following the exclusion of participants who were (1) under the age of 65, (2) lacking essential demographic information such as sex, age, and residence, and (3) missing critical variables including diet, smoking, drinking, exercise, and sleep, data of 12,766 participants were retained for analysis in this study.

### 2.2 Basic demographic data

The basic demographic information extracted for this study included gender, age, residence, years of education, co-residence, and self-assessed economic status. Residence was classified into three categories: city, town, or rural area. Co-residence was categorized as living with family, living alone, or residing in an institution. Self-perceived economic status was divided into five levels: very rich, rich, so-so, poor, and very poor. The handling of missing data differed by variable: the Expectation–Maximization (EM) algorithm was used for years of education, while multinomial logistic regression within a multiple imputation framework was applied to co-residence status and self-perceived economic status.

### 2.3 Health risk behaviors

Drawing upon previous research (1, 5, 19), we collected data on seven specific health risk behaviors, each of which were categorized using dichotomous variables.

#### 2.3.1 Inadequate sleep

Sleep duration was assessed using the question “How many hours do you normally sleep?” Based on relevant research (20), sleep duration of less than six hours is considered inadequate sleep.

#### 2.3.2 Insufficient fruit intake

Fruit consumption was evaluated using the multiple-choice question “Do you eat fresh fruit?,” with four response options: A. every day or almost every day; B. quite often; C. occasionally; D. rarely or never. Responses of C or D are considered insufficient fruit intake (7, 21).

#### 2.3.3 Insufficient vegetable intake

Vegetable consumption was assessed using a method similar to that for fruit intake. Responses of C or D are similarly considered insufficient vegetable intake.

#### 2.3.4 Salty diet

Participants who selected ‘Salty’ in response to the question, “What kind of flavor do you mainly prefer?” were considered to have a salty diet (22).

#### 2.3.5 Smoking

Participants who answered ‘yes’ to the question, “Do you smoke at the present time?” were considered to exhibit smoking behavior.

#### 2.3.6 Drinking

Participants who answered ‘yes’ to the question, “Do you drink alcohol at the present time?” were considered to have drinking behavior.

#### 2.3.7 Irregular exercise

Participants who responded “no” to the question “Do you currently engage in regular exercise?” were considered to exhibit irregular exercise behavior.

### 2.4 Health outcomes

#### 2.4.1 Self-assessed quality of life

The self-assessed quality of life was measured using the multiple-choice question, “How do you rate your life at present?” with five response options: A. Very good; B. Good; C. Average; D. Poor; E. Very poor. Scores were assigned from 5 to 1, respectively.

#### 2.4.2 Self-assessed health level

The self-assessed health level was determined using a multiple-choice question, “How do you rate your health at present?” It employed the same response options and scoring system as the self-assessed quality of life measure.

### 2.5 Data analysis

Microsoft Excel 2021 and SPSS version 26.0 were used for the extraction, transformation, and coding of variables as required. Categorical variables are presented as frequencies and percentages. Continuous variables were assessed for normality using the Kolmogorov–Smirnov test and reported as mean ± standard deviation (M ± SD) if they followed a normal distribution; otherwise, they were expressed as median with interquartile range [M (P25, P75)]. Spearman correlation analysis was conducted to investigate the relationship between the number of health risk behaviors and health outcomes. Binary logistic regression was utilized to examine the demographic factors influencing the co-occurrence of health risk behaviors. All statistical tests were two-sided, with a significance level set at *p* < 0.05.

The observed-to-expected (O/E) ratio is used to evaluate the conditional probability of co-occurrence between two behaviors, serving as an important indicator for assessing the clustering risk among behaviors. The O/E ratio is an intuitive and robust measure of association for binary variables, reflecting whether health risk behaviors co-occur by chance (23). An O/E ratio greater than 1 indicates that two behaviors tend to co-occur, suggesting a clustering risk among health risk behaviors. The larger the O/E ratio, the stronger the clustering risk. The observed-to-expected (O/E) ratio for the co-occurrence of behavior A and behavior B was calculated as follows:


O/E=P(A∩B)/[P(A)×P(B)]

Where: P(A∩B) is the observed joint probability (i.e., the proportion of the sample exhibiting both behaviors). P(A) and P(B) are the marginal probabilities of behavior A and behavior B, respectively.

Network analysis was conducted using R (version 4.5.1). We applied the Ising model to estimate the network structure of health risk behaviors among older adults—a widely used approach for constructing networks from binary data. The network was fitted using the IsingFit package and visualized with the qgraph package. Topological structure indices of the network were calculated using the igraph package. Network stability was evaluated using the bootnet package, which performs 1,000 bootstrap replications to estimate the stability of edge weights via the case-dropping bootstrap method. Community detection was carried out using the classical Louvain algorithm, implemented in the igraph package, to identify clusters of densely interconnected behaviors. This allows for the detection of distinct patterns of behavioral co-occurrence within the health risk behavior network (24).

## 3 Results

### 3.1 Demographic characteristics

A total of 12,766 participants were included in this study for data analysis, with a median age of 84 (75, 93) years, consisting of 5,891 males and 6,975 females. Detailed demographic characteristics are presented in Table 1.

**Table 1:** Demographic characteristics.

Variables	*n* (%)
Gender	
Male	5,891 (46.15)
Female	6,875 (53.85)
Age (years)	
65 ~ 74	3,026 (23.70)
75 ~ 84	3,709 (29.05)
85 ~ 94	3,197 (25.04)
≥100	2,834 (22.20)
Residence	
City	2,854 (22.36)
Town	4,263 (33.39)
Rural	5,649 (44.25)
Years of education	
0	5,176 (40.55)
1 ~ 6	3,644 (28.54)
7 ~ 9	1,114 (8.73)
10 ~ 12	653 (5.12)
≥13	395 (3.09)
Miss	1784 (13.97)
Co-residence	
With household member(s)	10,165 (79.63)
Alone	2064 (16.17)
In a nursing home	384 (3.01)
Miss	153 (1.20)
Economic status	
Very rich	332 (2.60)
Rich	2,178 (17.06)
So so	8,870 (69.48)
Poor	1,120 (8.77)
Very poor	160 (1.25)
Miss	106 (0.83)

### 3.2 Co-occurrence characteristics of behaviors

**Figure 1:**
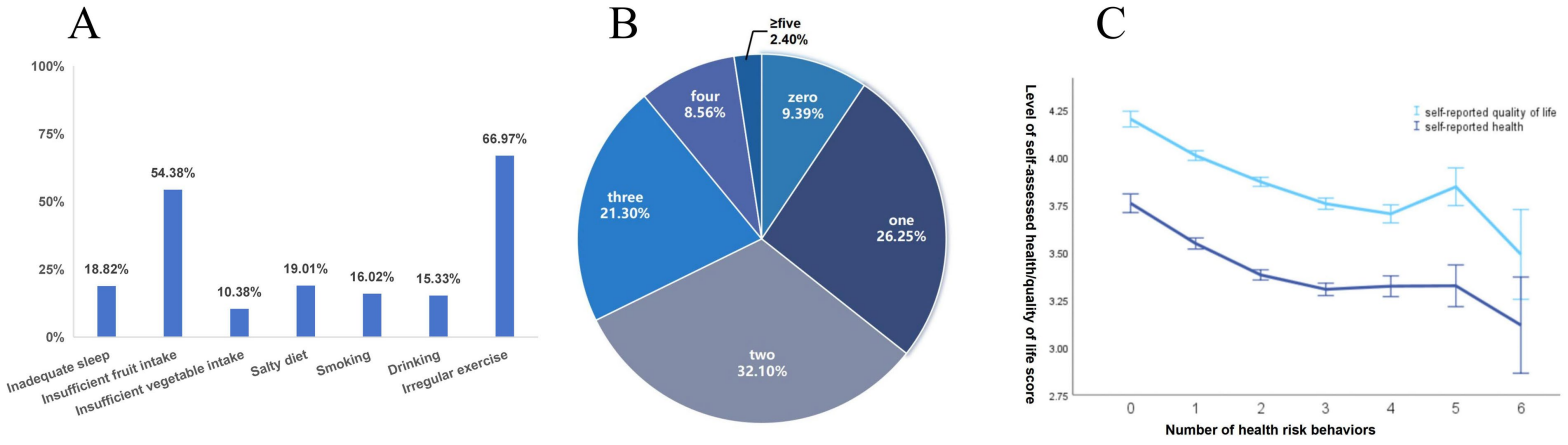
**(A)** Prevalence of health risk behaviors. **(B)** Number of co-occurrence of health risk behaviors in subjects. **(C)** Trends in participants’ Self-assessed health level and self-assessed quality of life with the number of co-occurrence health risk behaviors.

**Figure 2:**
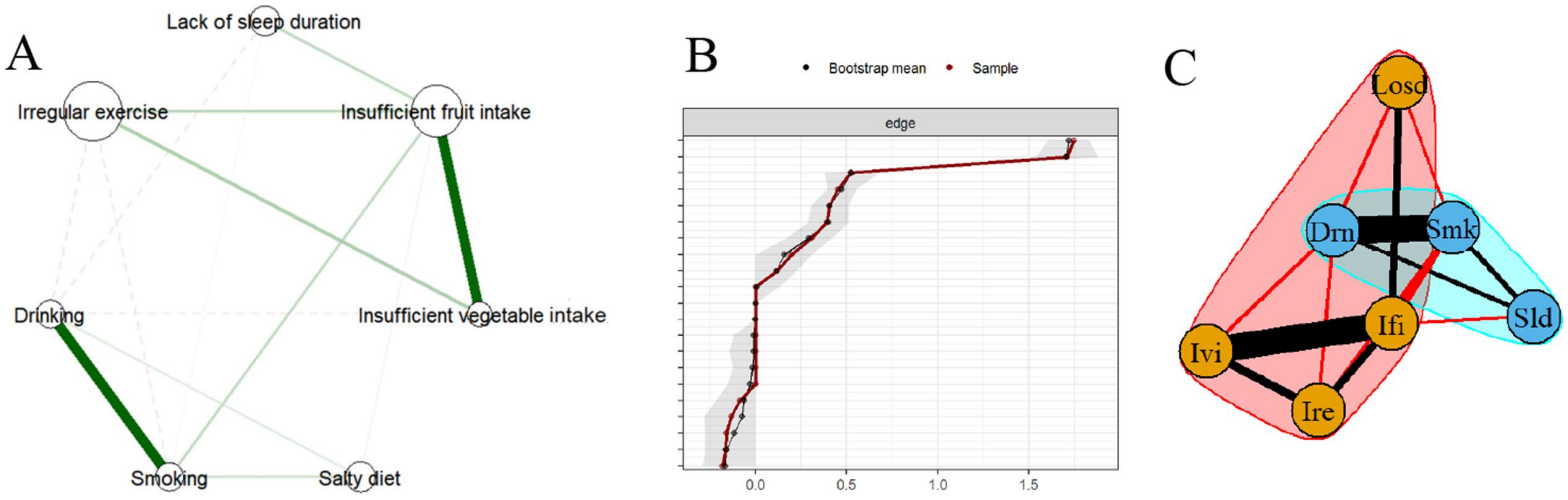
**(A)** The clustering network of health risk behaviors. **(B)**. Edge stability test of the network. **(C)** Community structure detected by the Louvain algorithm. Losd, lack of sleep duration; Ivi, insufficient vegetable intake; Ifi, insufficient fruit intake; Ire; irregular exercise, Drn, drinking; Smk, smoking; Sld, salty diet.

Figure 1 illustrates the prevalence of these health risk behaviors. Among the participants, irregular exercise was the most prevalent health risk behavior, while insufficient vegetable intake was the least prevalent. The co-occurrence of these behaviors is depicted in Figure 2. The majority (64.36%) exhibited two or more health risk behaviors. Spearman correlation analysis revealed that the number of co-occurring health risk behaviors was negatively correlated with self-assessed quality of life (*r* = −0.173, *p* < 0.001) and self-assessed health level (*r* = −0.141, *p* < 0.001), as shown in Figure 2B.

We conducted a binary logistic regression analysis using individuals with either zero or one health risk behavior as the control group to investigate the demographic factors influencing the co-occurrence of health risk behaviors. The findings indicated that males, residing in town or rural areas, older in age, with fewer years of education, living alone, and with lower economic status were more likely to experience co-occurrence of health risk behaviors. These results are detailed in Table 2.

**Table 2:** Logistic regression analysis between demographic factors and health risk behavior co-occurrence.

Variables	OR (95%CI)	*P*
Gender		
Male	Reference	
Female	1.86 (1.71 ~ 2.02)	<0.001
Age (per decade)	1.15 (1.11 ~ 1.19)	<0.001
Years of education		
0	Reference	
1 ~ 6	0.79 (0.72 ~ 0.87)	<0.001
7 ~ 9	0.67 (0.58 ~ 0.77)	<0.001
10 ~ 12	0.51 (0.43 ~ 0.61)	<0.001
≥13	0.46 (0.37 ~ 0.58)	<0.001
Residence		
City	Reference	
Town	2.30 (2.07 ~ 2.56)	<0.001
Rural	2.42 (2.19 ~ 2.69)	<0.001
Co-residence		
With household member(s)	Reference	
Alone	1.18 (1.05 ~ 1.31)	0.003
In a nursing home	0.96 (0.77 ~ 1.20)	0.739
Economic status		
Very poor	Reference	
Poor	0.74 (0.46 ~ 1.20)	0.219
So so	0.35 (0.22 ~ 0.55)	<0.001
Rich	0.21 (0.14 ~ 0.34)	<0.001
Very rich	0.22 (0.13 ~ 0.36)	<0.001
Constant	1.78	

### 3.3 Clustering characteristics of health risk behaviors

Table 3 presents the top 10 combinations of health risk behaviors based on O/E ratios; smoking and drinking demonstrate the highest clustering risk. Table 4 displays the top 10 behavior combinations ranked by O/E ratios for males and females separately. While the rankings for males and females showed considerable consistency, there were significant discrepancies in O/E ratios and incidence rates for certain identical behavior combinations.

**Table 3:** Ranking of participants’ health risk behavior combinations in terms of O/E ratios.

Rank	Combination	O/E (95%CI)	n (%)
1	④ + ⑤	2.67 (2.49 ~ 2.85)	837 (6.65)
2	① + ②	1.60 (1.51 ~ 1.69)	1,151 (9.02)
3	③ + ④	1.32 (1.21 ~ 1.44)	515 (4.03)
4	③ + ⑤	1.26 (1.15 ~ 1.37)	469 (3.76)
5	② + ⑥	1.20 (1.13 ~ 1.28)	1,068 (8.37)
6	① + ⑦	1.17 (1.11 ~ 1.23)	1,525 (11.95)
7	② + ⑦	1.16 (1.03 ~ 1.29)	289 (2.26)
8	① + ④	1.14 (1.08 ~ 1.21)	1,271 (9.96)
9	① + ⑥	1.08 (1.07 ~ 1.20)	5,039 (39.47)
10	① + ③	1.07 (1.07 ~ 1.13)	1,408 (11.03)

① Insufficient fruit intake; ② Insufficient vegetable intake; ③ Salty diet; ④ Smoking; ⑤ Drinking; ⑥ Irregular exercise; ⑦ Inadequate sleep.

**Table 4:** Ranking of O/E ratios for health risk behavior combinations for male and female participants.

	Male (*n* = 5,891)			Female (*n* = 6,875)		
Rank	Combination	O/E (95%CI)	*n* (%)	Combination	O/E (95%CI)	*n* (%)
1	④ + ⑤	1.66 (1.54 ~ 1.77)	768 (13.04)	④ + ⑤	4.07 (3.11 ~ 5.03)	69 (1.00)
2	① + ②	1.55 (1.41 ~ 1.69)	484 (8.22)	① + ②	1.63 (1.51 ~ 1.76)	667 (9.86)
3	② + ⑥	1.25 (1.14 ~ 1.37)	439 (7.45)	③ + ④	1.52 (1.18 ~ 1.86)	76 (1.10)
4	③ + ④	1.18 (1.07 ~ 1.29)	439 (7.45)	③ + ⑤	1.33 (1.06 ~ 1.60)	92 (1.33)
5	① + ⑦	1.17 (1.08 ~ 1.27)	603 (10.24)	① + ⑦	1.16 (1.09 ~ 1.24)	922 (13.37)
6	② + ⑦	1.15 (0.93 ~ 1.37)	104 (1.77)	② + ⑥	1.16 (1.07 ~ 1.25)	629 (9.12)
7	③ + ⑤	1.14 (1.03 ~ 1.26)	377 (6.40)	② + ⑦	1.15 (0.98 ~ 1.31)	185 (2.68)
8	① + ④	1.14 (1.07 ~ 1.21)	1,098 (18.64)	① + ④	1.10 (0.94 ~ 1.27)	173 (2.51)
9	① + ⑥	1.10 (1.05 ~ 1.14)	2,179 (36.99)	① + ⑥	1.07 (1.03 ~ 1.11)	2,860 (41.49)
10	① + ③	1.08 (1.00 ~ 1.16)	741 (12.58)	① + ⑤	1.04 (0.90 ~ 1.17)	226 (3.27)

① Insufficient fruit intake; ② Insufficient vegetable intake; ③ Salty diet; ④ Smoking; ⑤ Drinking; ⑥ Irregular exercise; ⑦ Inadequate sleep.

Figure 2 illustrates the clustering network of health risk behaviors, where the size of each node corresponds to the prevalence of the respective behaviors, and the thickness of the edges represents the strength of association between pairs of behaviors. Topological analysis indicates that the network exhibits high connectivity, with a density of 0.667 and a mean strength of 1.467. Moreover, the network demonstrates a strong tendency for clustering, as reflected by a clustering coefficient of 0.533. According to the overall network edge weight stability analysis results (Figure 2B), the 95% confidence interval for the edge weight values is relatively narrow. Therefore, it indicates that the estimation of the overall network edge weight values is accurate.

The Louvain algorithms indicate the presence of two distinct sub-communities within the network, as shown in Figure 2C. Consequently, based on the community segmentation results, we categorized the health risk behaviors into two clustering patterns: the addictive behavior pattern, encompassing smoking, drinking, and salty diet, and the unhealthy activity-eating pattern, which includes insufficient vegetable intake, insufficient fruit intake, inadequate sleep, and irregular exercise.

## 4 Discussion

This study examined the co-occurrence and clustering characteristics of seven health risk behaviors among older adults in China. We found that 64.36% of the participants engaged in two or more health risk behaviors, a prevalence rate that aligns with findings from earlier studies (5, 6, 8, 15, 25). Furthermore, the number of co-occurring health risk behaviors was negatively associated with the participants’ self-rated health and overall quality of life. These results highlight the significant prevalence of co-occurring health risk behaviors in this demographic, underscoring a critical concern that may adversely affect health outcomes.

Factors such as being male, residing in townships or rural areas, being older, having fewer years of education, living alone, and having lower economic status were linked to a higher likelihood of engaging in co-occurring health risk behaviors, corroborating previous research findings (25–27). The findings of this study underscore the critical importance of prioritizing interventions targeting the specified population groups. Furthermore, they emphasize the necessity of comprehensively exploring the social determinants that underpin these factors to develop more effective prevention strategies. Given the unique characteristics of these high-risk populations, future interventions must account for their complex social and personal circumstances. For instance, there is a need to enhance primary healthcare access and quality, strengthen community support for elders living alone, and bolster economic aid and social welfare to counter poverty’s impact on health.

Our analysis of clustering characteristics revealed that the O/E ratios for certain behavior pairs exceeded 1. The findings from the O/E analysis were further substantiated by topological analysis, which identified high network density and average strength. These metrics collectively suggest that the seven health-risk behaviors are not independent but form a tightly interconnected cluster, functioning as a cohesive “behavioral syndrome” in older adults. This implies that among older adults, if an individual engages in one health-risk behavior, they are highly likely to exhibit multiple other health-risk behaviors simultaneously. The high clustering coefficient and the results of community detection in the network analysis further indicate that these behaviors tend to form specific, intricately interconnected clusters. Taken together, these findings point to the promise of developing integrated, multi-behavior intervention strategies that target the observed clustering patterns. Such holistic approaches may yield greater benefits for older adults than those focusing on single behaviors in isolation.

Furthermore, the clustering characterization results revealed two interesting findings. First, a significant clustering risk was identified among the salt diet, smoking, and drinking; these factors together form a clustering pattern. Due to sociocultural influences, cross-tolerance, and neurobiological mechanisms, smoking and drinking are considered to be highly correlated (28, 29). This linkage is particularly evident in China, where offering cigarettes and urging alcohol drinking serve as important rituals for establishing and maintaining social relationships, thereby reinforcing the clustering of smoking and drinking. Smoking and drinking can significantly diminish taste sensitivity (30, 31), and alcohol consumption can also disrupt hunger signals, potentially leading to increased consumption of unhealthy salty snacks (32). Additionally, akin to alcohol and tobacco, salt (sodium chloride) exhibits addictive properties (33). These elements collectively may facilitate the close association and clustering of these three behaviors, which is also supported by the results of a community-based population survey from Korea (34).

Smoking, alcohol consumption, and excessive salt intake are established risk factors for numerous chronic diseases, including hypertension, stroke, and cardiovascular disease, thereby constituting a primary focus in the health management of older adults. Our findings suggest that public health strategies should consider the interrelationship between these behaviors. For example, an older adult who smokes or drinks alcohol may also be at high risk for excessive salt intake. Thus, it is important to assess and intervene in their diet.

Secondly, while the co-occurrence rate of smoking and drinking among older women is lower than that among older men (1% vs. 13.04%), the clustering risk for older women is significantly higher than that for men (O/E = 4.07 vs. O/E = 1.66). This pattern is also evident in other behavioral combinations, such as a salt diet combined with drinking or smoking. One possible explanation for this phenomenon is sex differences in the reward system. Empirical evidence suggests that nicotine and alcohol together lessen the relaxation effects of either substance in men but enhance the sense of relaxation in women (35). Additionally, Liu’s study indicates that in England, females who drink heavily are less likely to attempt to quit smoking, but males are more likely to attempt quitting in England (36). This finding aligns with the results of our study.

Of concern, evidence suggests that the health effects of smoking and alcohol consumption may be more detrimental to women than to men. Our previous research indicated that smoking was significantly associated with a higher prevalence of cardiometabolic diseases among females compared to males (37). Furthermore, extensive laboratory and population-based studies have demonstrated that women are more susceptible than men to the toxic effects of alcohol (38, 39). In addition, a study by Li et al. (40) found that women who both smoke and drink have a 1.54 times greater risk of developing heart disease compared to those who do not engage in these behaviors, whereas no statistically significant difference is observed in men. Therefore, given the increasing rates of tobacco and alcohol use among women (41), there is an urgent need to develop gender-sensitive interventions specifically tailored to address smoking and drinking behaviors in this population.

This study has several limitations. First, the use of cross-sectional survey data restricts our ability to draw causal inferences. Second, reliance on self-reported variables may introduce bias. Thirdly, specific to dietary assessment, the data on vegetable and fruit consumption captured only frequency, not quantity, while the behavior of having a salty diet was defined by self-reported flavor preference, potentially affecting the accuracy of our behavioral risk evaluation. Finally, despite the inclusion of a nationally representative sample, the findings may not be generalizable to other populations, such as those in Europe or the United States.

## 5 Conclusion

In older adults of China, co-occurring health-risk behaviors are a prevalent issue. The number of these co-occurring behaviors is inversely associated with both self-rated health status and self-rated quality of life. Health-risk behaviors exhibit a clustering effect and demonstrate distinct clustering patterns. Public health strategies should account for the co-occurrence and clustering characteristics of health behaviors in older adults, thereby facilitating the development of targeted interventions for managing multiple behaviors.

## Data Availability

Publicly available datasets were analyzed in this study. This data can be found at: https://doi.org/10.18170/DVN/WBO7LK.
